# Therapeutic Effects of HIF-1α on Bone Formation around Implants in Diabetic Mice Using Cell-Penetrating DNA-Binding Protein

**DOI:** 10.3390/molecules24040760

**Published:** 2019-02-20

**Authors:** Sang-Min Oh, Jin-Su Shin, Il-Koo Kim, Jung-Ho Kim, Jae-Seung Moon, Sang-Kyou Lee, Jae-Hoon Lee

**Affiliations:** 1 Department of Prosthodontics, College of Dentistry, Yonsei University, 134 Shinchon-dong, Seodaemoon-gu, Seoul 03722, Korea; smtop38@naver.com; 2 Department of Biotechnology, College of Life Science and Biotechnology, Yonsei University, 134 Shinchon-dong, Seodaemoon-gu, Seoul 03722, Korea; jinsuand@naver.com (J.-S.S.); tasada19@hanmail.net (I.-K.K.); jsmoon4@hanmail.net (J.-S.M.); sjrlee@yonsei.ac.kr (S.-K.L.); 3 Research Institute for Precision Immuno-medicine, Good T Cells Incorporated, 134 Shinchon-dong, Seodaemoon-gu, Seoul 03722, Korea; jhokim@goodtcells.co.kr

**Keywords:** diabetes mellitus, hypoxia-inducible factor-1α, angiogenesis, bone formation, osteogenesis, protein transduction domain

## Abstract

Patients with uncontrolled diabetes are susceptible to implant failure due to impaired bone metabolism. Hypoxia-inducible factor 1α (HIF-1α), a transcription factor that is up-regulated in response to reduced oxygen during bone repair, is known to mediate angiogenesis and osteogenesis. However, its function is inhibited under hyperglycemic conditions in diabetic patients. This study thus evaluates the effects of exogenous HIF-1α on bone formation around implants by applying HIF-1α to diabetic mice and normal mice via a protein transduction domain (PTD)-mediated DNA delivery system. Implants were placed in the both femurs of diabetic and normal mice. HIF-1α and placebo gels were injected to implant sites of the right and left femurs, respectively. We found that bone-to-implant contact (BIC) and bone volume (BV) were significantly greater in the HIF-1α treated group than placebo in diabetic mice (*p* < 0.05). Bioinformatic analysis showed that diabetic mice had 216 differentially expressed genes (DEGs) and 21 target genes. Among the target genes, NOS2, GPNMB, CCL2, CCL5, CXCL16, and TRIM63 were found to be associated with bone formation. Based on these results, we conclude that local administration of HIF-1α via PTD may boost bone formation around the implant and induce gene expression more favorable to bone formation in diabetic mice.

## 1. Introduction

Dental implants have become an efficient and predictable treatment for replacing missing teeth. The number of implants placed in the United States has been steadily increasing at 12% annually, with improvements in implant materials, designs, and surgical techniques [1]. Despite an implant success rate of 95% in the general population [2], certain risk factors may predispose individuals to implant failure [3]. Among various patient-related risk factors, poorly controlled diabetes mellitus, a chronic metabolic disease characterized by hyperglycemia, has been considered a relative contraindication to dental implant [4,5,6].

Implant success is highly dependent on osseointegration, the process in which bone and implant surface become structurally and functionally integrated without interposition of the non-bone tissue layer [7]. Osseointegration, which involves bone repair and remodeling, critically affects implant stability [8]. However, the hyperglycemic condition of diabetes inhibits osteoblastic differentiation, mineralization, and adherence of the extracellular matrix and stimulates bone resorption, all consequently interfering with wound healing and bone regeneration [9,10]. Previous experimental studies have reported decreased bone-to-implant contact (BIC) and delayed new bone formation around the implant in diabetic animal models, proving that hyperglycemia impairs osseointegration [11].

Numerous studies have demonstrated that a chronic high glucose level results in defective responses of tissues to hypoxic conditions by impairing the function of hypoxia-inducible factor 1α (HIF-1α) [12,13,14]. Transcription factor HIF-1α is up-regulated in response to reduced oxygen conditions and influences numerous target genes, such as vascular endothelial growth factor (VEGF) and runt-related transcription factor 2 (RUNX2), which are known to be associated with angiogenesis and osteogenesis [15,16,17]. HIF-1α, which is well known to play a pivotal role in wound healing, is stabilized against degradation and transactivates under hypoxia [15]. A study carried out by Zou et al. demonstrated that osteogenesis and angiogenesis were enhanced around implants by the up-regulation of HIF-1α in rat bone mesenchymal stem cells (BMSCs) in animal models [18]. In addition, previous studies investigating the effects of HIF-1α on bone regeneration showed that the functions of osteoblasts and chondrocytes are directly regulated by HIF-1α during bone fracture healing in animal models [19].

As many studies have associated the malfunction of HIF-1α in diabetic animal models with delayed bone recovery, attempts have been made to improve bone healing by applying HIF-1α. However, the application of HIF-1α using mesenchymal stem cells to increase its expression is inefficient and time-consuming. To maximize the efficiency of delivery to the implant site, a protein transduction domain (PTD)-mediated DNA delivery system was used in this study. PTDs are short peptides that efficiently transport various proteins, nucleic acids, and nanoparticles into cells across the plasma membranes. The low toxicity and high transduction efficiency of this protein-based strategy constitutes a beneficial method for delivering target DNA to the nucleus [20]. Indeed, our recent study showed that the overexpression of HIF-1α induced by the PTD-mediated DNA delivery system resulted in an increased expression of VEGF and angiogenesis in vitro and in vivo [21].

In this study, taking advantage of the fact that PTD can deliver HIF-1α into cell nuclei, we designed an experiment to determine whether local application of HIF-1α into the implanted sites by using PTD in the femur of diabetic mice enhances osseointegration compared with placebo controls. Using RNA sequencing and histomorphometric analysis, we observed new bone formation and significant changes in the expression of genes associated with wound healing.

## 2. Results

### 2.1. RNA Sequencing and Differentially Expressed Genes (DEGs)

Different combinations of groups were designed and RNA sequencing was performed to identify DEGs. Group NH, normal mice with HIF-1α gel; group NP consisted of normal mice with placebo gel; group DH, diabetic mice with HIF-1α gel; group DP, diabetic mice with placebo gel.

The number of up- and down-regulated genes with a certain cutoff (2-fold; *p*-value < 0.05; FDR < 0.1) for all combinations are described in Table 1. A total of 216 genes were differentially expressed in the DH group compared to the DP group. On the other hand, there were 95 DEGs in the case of normal mice.

### 2.2. Target Genes of HIF-1α in Bioinformatic Analysis

The software program, TRANSFAC^®^ (Qiagen N.V., Valencia, CA, USA), was used to select the target genes of HIF-1α. Twenty-one genes were identified as target genes of HIF-1α in diabetic mice (Table 2). Among the 21 detected genes, NOS2, GPNMB, CCL2, CCL5, CXCL16, and TRIM63 were found to be associated with wound healing or bone healing-related genes. The functions of these genes are described in Table 3 [22,23,24,25,26,27]. In normal mice, five genes (NOS2, CCL2, CCL5, CD274, TNF) were identified as target genes of HIF-1α.

### 2.3. Histologic Analysis

**Figure 1:**
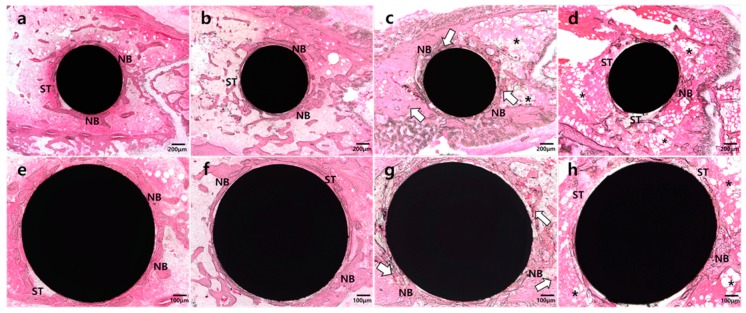
Representative images of undecalcified specimens of four groups. (**a**) and (**e**): Abundant and well-developed new bone (NB) formation observed around the implant in the normal mice with HIF-1α gel (NH) group, with some soft tissue (ST) engagement observed; (**b**) and (**f**): Thin and well-defined new bone formation observed in the normal mice with placebo gel (NP) group; (**c**) and (**g**): Plentiful vascular sinusoids with red blood cells (white arrow) and newly-formed bone surrounding the implant in the diabetic mice with HIF-1α gel (DH) group; (**d**) and (**h**): Fibrotic and adipose tissue (asterisk) surrounding the implant in the diabetic mice with placebo gel (DP) group.

In the NH group (Figure 1a,e), abundant and smooth-lined mature bone formation was observed. Mature and smooth-lined bone was also observed in the NP group (Figure 1b,f), but in a lesser amount than in the NH group. In contrast, most of the implant surface in the DP (Figure 1d,h) group showed soft tissue attachment and abundant adipose tissue in surrounding areas. Moreover, bone formation was irregular. In the DH group (Figure 1c,g), many vascular sinusoids with red blood cells were located around implants, and more bone formation and attachment around implants were observed than in the DP group. In addition, there was a tendency toward increased bone formation at the HIF-1α application site around implants. Bone marrow was filled with adipose tissue in areas distant from the application site.

### 2.4. Histomorphometric and Statistical Analysis

**Figure 2:**
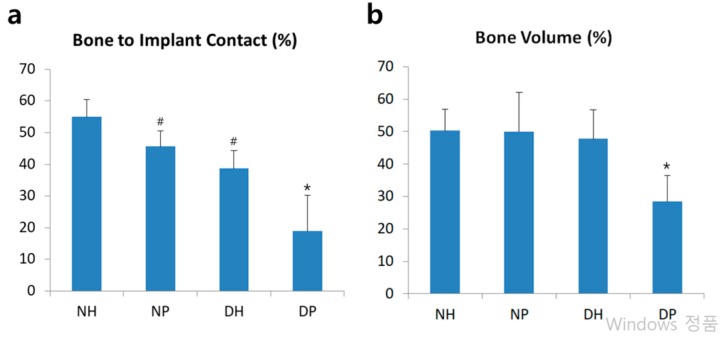
Histomorphometric analysis. (**a**): Linear percentage of direct BIC in the total surface of implants. (**b**): Percentage of newly formed bone area in the circumferential zone within 100 μm of the implant surface. Data represent mean ± SD. *: *p*-value < 0.05 vs all other groups; #: *p* < 0.05 vs NH group.

BIC was observed in all specimens. All groups in this study demonstrated normality in the parametric test (Shapiro-Wilk test). The BIC of the HIF-1α treated groups was significantly higher than that of placebo groups in both normal and diabetic mice. There was no significant BIC difference between the NP and DH groups (Figure 2a). Among the diabetic mice, the DH group showed significantly greater BV than the DP group while the groups of normal mice did not show any significant differences in BV. Only the DP group showed significantly lower BV among the four groups (Figure 2b).

## 3. Discussion

Previous studies reported that hyperglycemia, even in hypoxic conditions, negatively affects the stability and activation of HIF-1α and inhibits the expression of target genes of HIF-1α, which are critical to wound healing [10]. On the other hand, it has been reported that the exogenous increase of HIF-1α resulted in improved bone regeneration and osseointegration around the implant in normoglycemic conditions [18,19]. This study was designed to test the hypothesis that the exogenous increase of HIF-1α would improve bone regeneration in diabetic mice because, based on previous studies, endogenous HIF-1α was suppressed in a hyperglycemic environment and failed to function.

In this study, we found that: (1) HIF-1α improves bone formation around the implant in diabetic mice; (2) HIF-1α induces gene expression that is more favorable to bone regeneration; and (3) exogenous HIF-1α has a greater effect on diabetic mice than normal mice.

Histologic results show that adipose and soft tissue were more engaged in diabetic mice femur bone marrow than in normal ones. Remarkably, most of the bone marrow in diabetic mice was composed of adipose tissue. In addition, the thickness and amount of regenerated bone was thinner and less in diabetic mice. The bone shape was highly irregular and fragile. The histological state of diabetic mice specimens was unfavorable for implant maintenance. These results were coincident with previous studies, which reported slower bone healing in diabetic mice than normal mice as well as poorer biomechanical and histologic bone quality after initial healing [10,28].

Histomorphometric results showed that the DH group had greater bone contact and volume than the DP group. Based on histologic specimens, more vascular sinusoids were generated in those groups with HIF-1a application, demonstrating that HIF-1α increased the expression of VEGF and improved angiogenesis as in our previous study, in which HIF-1α was applied in the same way as this study [21]. It is speculated that HIF-1α, which enhanced angiogenesis, also enhanced bone regeneration and increased BIC and BV levels. In addition, distant sites were full of adipose tissue compared to HIF-1α application sites near the implant, which consisted mainly of dense bone. The difference was even more evident in comparison with the DP group, in which tissue around the implant was filled with adipose tissue. There was no significant difference in BIC and BV when comparing the DH and NP groups. We thus expect diabetic mice to have as much bone formation as normal mice when HIF-1α is applied.

Bioinformatic analysis showed that diabetic mice had 216 DEGs and 21 target genes whereas normal mice had 95 DEGs and 5 target genes. Moreover, the DEGs and target genes in normal mice were mostly included in those of diabetic mice. These results suggest that the application of HIF-1α, suppressed in hyperglycemic conditions, activated the expression of HIF-1α target genes. On the other hand, HIF-1α target genes were less activated in normal conditions because endogenous HIF-1α was already sufficiently expressed around the injured tissue. It is therefore assumed that genes downstream of HIF-1α were more actively expressed in diabetic mice when exogenous HIF-1α was provided, which may account for the histomorphometric results of improved BIC and BV.

In normal conditions, HIF-1α also improved BIC, although not significantly, consistent with previous studies [15,18]. However, HIF-1α was more effective in diabetic mice in that the DH group showed significantly increased BIC and BV than did DP while the NH group showed no significant difference in BIC and BV compared to the NP group. Based on this result, it is assumed that exogenous HIF-1α worked more effectively in hyperglycemic conditions than normoglycemic conditions.

Previous studies used mesenchymal stem cells to increase expression of HIF-1α, a process considered inefficient due to the long and complicated preparation [18,29]. On the other hand, the PTD-mediated DAN delivery system used in this study is a simple and efficient method for the application of HIF-1α expression plasmid, which can easily be produced in large quantities and injected. Moreover, the molecular complex containing Hph-1-GAL4-DBD and HIF-1α-UAS can be solidified into gel form for applications to a local site without diffusion. The efficacy of HIF-1α delivery with a PTD-mediated system in vivo and in vitro was shown in our previous study [21], wherein PTD-mediated HIF-1α delivery increased HIF-1α, VEGF, and other HIF-1α target genes in vitro and in vivo. However, additional studies are needed to determine what the target cells are, how osteoblast progenitors and osteoclasts respond, and whether the gel form releases HIF-1α ideally or not. Moreover, future studies on higher mammals, such as dogs and pigs, may clarify the effects of HIF-1α.

Based on this study, we would like to suggest that the use of angiogenic growth factors, such as HIF-1α, rather than osteogenic growth factors, like BMP, could improve bone quality and quantity around the implant. Bone regeneration can be enhanced by applying bone morphogenetic factor (BMP), an osteogenic growth factor, or vascular endothelial growth factor (VEGF), an angiogenic growth factor [30]. The administration of BMP has been reported to improve bone regeneration in numerous dental studies and is practiced in the dental field [31]. However, several complications associated with BMP treatment have been reported, such as uncontrolled release rates, a short period of BMP release, and a high initial burst of release [32]. BMP also causes an unexpected immune reaction, spontaneous swelling of soft tissues, and difficulties in controlling the diffusion [33]. We think that HIF-1α may serve as an alternative to BMP, which presents several disadvantages.

## 4. Materials and Methods

### 4.1. Ethics Statements

This study was carried out in accordance with the guidelines established by the Laboratory Animal Care and Use Committee at Yonsei University Biomedical Research Institute (2014-0032). All surgical procedures were performed via intraperitoneal injection, analgesia, and antibiotics being administered at appropriate time points to minimize suffering and pain. The ARRIVE Guidelines for reporting animal research were abided by in all sections of this report [34].

### 4.2. Animal Models

Thirteen 8-week-old male C57BL/6 mice (21 g) from Charles River (Orientbio, Gapyeong-gun, Korea) and 13 8-week-old male C57BLKS/J-db/db mice (38 g, Leptin-receptor deficient type 2 diabetes mice) from Charles River (Hinobreeding Center, Tokyo, Japan) were used for the experiments. They were maintained in the Avison Biomedical Research Center at Yonsei University College of Medicine at 23 ± 2 °C and 50 ± 10% humidity under 12 h of light alternating with 12 h of darkness.

### 4.3. Preparation of HIF-1α Gene Construct and Hph-1-GAL4 DNA Binding Domain Protein

HIF-1α encoded plasmid and Hph-1-GAL4 DNA binding protein were provided by Sang-Kyou Lee’s Laboratory at Yonsei University, Department of Biotechnology. The HIF-1α gene was inserted into pEGFP-N1 UAS plasmid containing five consensus GAL4 binding sites (UAS: CGGAGGACAGTACTCCG) (HIF-1α-UAS). The GAL4 DNA binding domain that encodes the DNA-interactive domain of yeast transcription factor GAL4 was cloned into pRSETB plasmid (Clonetech) expression vector containing Hph-1-PTD sequence (YARVRRRGPRP) at the N-terminus (Hph-1-GAL4-DBD). pRSETB plasmid with the Hph-1-GAL4 DNA binding domain was transformed into Escherichia coli BL21 star (DE3) pLysS strain (Invitrogen). Protein expression and purification were performed as described previously [20].

### 4.4. Preparation of HIF-1α Gel

**Figure 3:**
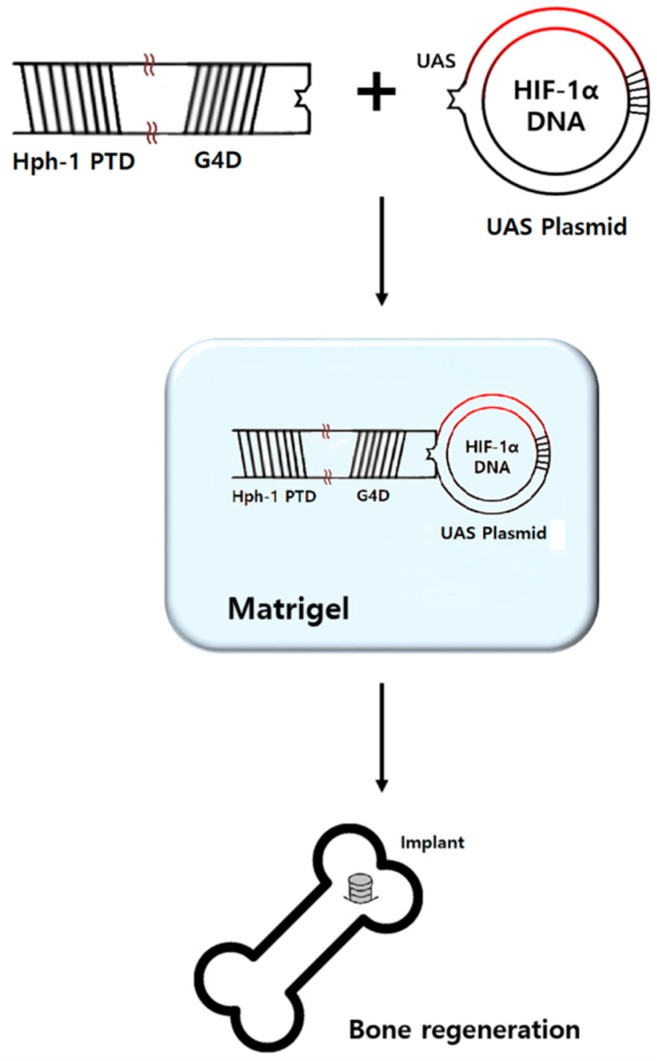
GAL4 DNA binding domain (G4D) was cloned into pRSETB plasmid expression vector containing Hph-1-PTD sequence at the N-terminus (Hph-1-G4D). The HIF-1α gene was inserted into pEGFP-N1 UAS plasmid containing five consensus GAL4 binding sites (HIF-1α-UAS plasmid). HIF-1α-UAS plasmid and Hph-1-G4D were mixed at a 1:50 mass ratio. The mixture and liquid form of Matrigel^®^ were blended at a 1:1 ratio just before application for bone regeneration.

One microgram of HIF-1α-UAS plasmid was mixed with 50 µg of Hph-1-GAL4-DBD at room temperature for 15 min right before surgery, as previously described [21]. The liquid form of Matrigel^®^ (BD Biosciences, San Jose, CA, USA) and the mixture were blended at a 1:1 ratio just before the application of HIF-1α gel during surgery (Figure 3). Pure Matrigel^®^ was used as a placebo gel. Matrigel^®^ was stored in a liquid state at a temperature of −72 °C in the freezer because it solidifies at 4 °C.

### 4.5. Surgical Procedure

**Figure 4:**
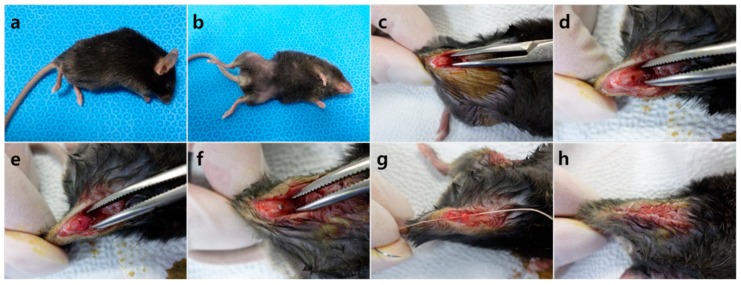
Surgical procedure of the implant. (**a**) Anesthetized mouse, (**b**) skin preparation, (**c**) incision, (**d**) preparation of the implant site, (**e**) gel injection, (**f**) placement of the implant, (**g**) closure of the surgical site layer by layer, (**h**) post surgery.

Thirteen C57BL/6 mice (21 g) and 13 C57BLKS/J-db/db mice (38 g) were given two weeks of acclimatization before surgery. Implant placing methods followed Xu et al. [35]. The mice were anesthetized by intraperitoneal injection of a mixture of Zoletil 50 (30 mg/kg, Vibac Laboratories, Carros, France) and Rompun (10 mg/kg, Bayer Korea, Seoul, Korea) (Figure 4a), the surgical site being shaved (Figure 4b) and disinfected with 10% polyvinylpyrrolidone iodine. An incision was made above both knee joints and the anterior-distal aspect of the femur was accessed using medial parapatellar arthrotomy (Figure 4c). Implant sites were prepared on the anterior-distal surface of the femur through sequential drilling with 0.5 mm and 0.9 mm round burs and 0.7 mm stainless steel twist drills at 1500 rpm with cooled sterile saline irrigation (Figure 4d). To effectively deliver HIF-1α to the implant site via local injection, gel phase materials were prepared as described. HIF-1α gel was injected to the preparation site and cancellous bone of the right femur, placebo gel being injected to the same areas for the left femur (Figure 4e). When the gel hardened, pure titanium implants with a machined surface (1 mm in diameter; 2 mm in length; Shinhung, Seoul, Korea) were inserted into the undersized hole with mild pressure (Figure 4f). The muscles and skin were sutured independently to cover and stabilize the implant (Figure 4g,h). Antibiotics were injected at fixed times daily for 3 days (Enrofloxacin 5 mg/kg, twice a day; Meloxicam, 1 mg/kg, once a day) [36,37]. Three C57BL/6 (21 g) and three C57BLKS/J-db/db (38 g) mice were sacrificed 4 days after the surgery for RNA sequencing, and ten mice from each strain were sacrificed two weeks after the surgery for histologic and histomorphometric analysis.

### 4.6. RNA Sequencing

Three mice from each strain (C57BL/6 (21 g) and C57BLKS/J-db/db (38 g)) were sacrificed and bone within 1 mm of the implant was taken for RNA sequencing analysis. Because factors related to bone formation are mostly expressed 4 days after implant surgery, RNA sequencing was performed at that time point [38].

RNA purity was determined by assaying 1 µL of total RNA extract on a NanoDrop8000 spectrophotometer (Thermo Fisher Scientific, Wilmington, DE, USA). Total RNA integrity was checked using an Agilent Technologies 2100 Bioanalyzer (Agilent Technologies, Foster City, CA, USA) with an RNA Integrity Number (RIN) value greater than 8. mRNA sequencing libraries were prepared according to the manufacturer’s instructions (Illumina TruSeq RNA Prep Kit v2, Illumina, San Diego, CA, USA). mRNA was purified and fragmented from total RNA (1 μg) using poly-T oligo-attached magnetic beads using two rounds of purification. Cleaved RNA fragments primed with random hexamers were reverse transcribed into first strand cDNA using reverse transcriptase and random primers. The RNA template was removed and a replacement strand was synthesized to generate double-stranded (ds) cDNA. End repair, A-tailing, adaptor ligation, cDNA template purification, and enrichment of the purified cDNA templates using PCR were then performed. The quality of the amplified libraries was verified by capillary electrophoresis (Bioanalyzer, Agilent Technologies, Foster City, CA, USA). After performing qPCR using SYBR Green PCR Master Mix (Applied Biosystems, Thermo Fisher Scientific, Foster City, CA, USA), we combined libraries that were index tagged in equimolar amounts in the pool. RNA sequencing was performed using the Illumina NextSeq 500 system (Illumina, San Diego, CA, USA) following the protocols provided for 2 × 75 sequencing.

Reads for each sample were mapped to the reference genome (mouse mm10) by Tophat (v2.0.13). The aligned results were added to Cuffdiff (v2.2.0) to report differentially expressed genes. Geometric and pooled methods were applied for library normalization and dispersion estimation.

### 4.7. Identification of DEGs

Of the various Cuffdiff output files, “gene_exp.diff” was used to identify DEGs. Two filtering processes were applied to detect DEGs between control and case groups. First, only genes having Cuffdiff status code “OK” were extracted. The status code indicates whether each condition contains enough reads in a locus for a reliable calculation of the expression level, “OK” indicating that the test was successful in calculating the gene expression level. For the second filtering, the 2-fold change was calculated and only genes belonging to the following range were selected:

Up-regulated:

log2[case] − log2[control] > log2(2) = 1(1)

Down-regulated:

log2[case] − log2[control] < log2(1/2) = −1(2)

### 4.8. Identification of Target Genes of HIF-1α

The software program, TRANSFAC^®^ (Qiagen N.V., USA), was used to select the target genes of HIF-1α. TRANSFAC^®^ provides not only a database of eukaryotic transcription factors, but also an analysis of transcription factor binding sites. MATCH analysis was performed with TRANSFAC^®^ using DEGs and an HIF-1α related matrix was selected from the results [39].

### 4.9. Histologic and Histomorphometric Analysis

Ten mice from each strain (C57BL/6 (21 g) and C57BLKS/J-db/db (38 g)) were sacrificed at 2 weeks after implant surgery to histologically evaluate mature bone in the healing process and estimate the implant stability in each group [37]. Femurs from both sides were obtained and fixed at 10% buffered formalin. After a week of fixation, they were embedded in light curing epoxy resin. The specimens were prepared with a cutting distance of 0.5 mm from the apical end of the implant. Sections were cut with a thickness of 50 μm via a grinding system, stained with hematoxylin and eosin (H&E), then observed using light microscopy (Leica DM LB, Wetzlar, Germany). IMT iSolution Lite ver 8.1^®^ (IMT i-Solution Inc., Vancouver, BC, Canada) was used for histomorphometric measurement. The BIC ratio was calculated as the linear percentage of direct BIC to the total surface of implants. The BV ratio was calculated as the percentage of newly formed bone area to a circumferential zone within 100 μm of the implant surface.

### 4.10. Statistical Analysis

All statistical procedures were performed using IBM SPSS 23.0 (IBM Corp., Armonk, NY, USA). Raw histomorphometric measurement data were used to calculate the mean ± SD. The Shapiro-Wilk test was used to test normality and one-way analysis of variance (ANOVA) was used to compare groups, which were considered independent. Post hoc was performed with Scheffe’s method. The value of *p* < 0.05 was considered statistically significant.

## 5. Conclusions

PTD-mediated delivery of HIF-1α into implant sites increases local HIF-1α levels, giving rise to a hyperglycemic environment that favors bone regeneration. This method holds tremendous potential and merits further study to determine its effectiveness as a local delivery system.

## Figures and Tables

**Table 1:** The number of differentially expressed genes (DEGs) in each combination.

2-Fold; *p*-Value < 0.05; FDR < 0.1	Up	Down
Group NH and NP	94	1
Group DH and DP	201	15

These genes were selected according to the cut-off (2-fold; *p*-value < 0.05; FDR < 0.1), HIF-1α treated group was compared to the placebo group.

**Table 2:** 21 target genes of Hypoxia-inducible factor 1α (HIF-1α) out of 216 DEGs in diabetic mice through TRANSFAC^®^.

Gene Symbol	Fold Change (log2X)	Molecule Type
CACNA1S	1.07	Calcium channel
CCL2	1.31	Chemokine
CCL5	1.49	Chemokine
CD274	1.70	Ligand
CXCL16	1.11	Chemokine
COBL	1.46	Cordon bleu
DES	1.20	Enzyme
GPNMB	1.06	ECM
IL2RA	1.76	Binding protein
JSRP1	1.05	Membrane protein
MARCO	2.88	Binding protein
MIA	−1.03	Structural protein
MURC	1.12	Structural protein
MYH14	1.14	Enzyme
MYL3	−1.54	Structural protein
NOS2	1.41	Enzyme
OAS2	1.21	Enzyme
PRKAG3	1.50	Protein kinase
TGTP1	2.16	GTPase
TRIM63	1.93	Ubiquitin protein ligase
TTN	1.05	ECM

With fold change, 1 indicates a two-fold increase in expression and −1 indicates a two-fold decrease in expression. Genes related to tissue healing or bone regeneration are in red.

**Table 3:** Target genes related to tissue healing or bone regeneration.

Gene Symbol	Functions of Target Gene
NOS2	Mediate increased blood flow Reparative collagen accumulation
GPNMB	Inducing differentiation and mineralization of hBMSCs into osteoblasts Increasing endothelial cell proliferation and migration, resulting in capillary tube formation
CXCL16	Recruitment of osteoclasts to restore the bone lost during the resorptive phase of bone turnover
CCL2	Consistent up-regulation during implant healing
CCL5	Promoting neovascularization and eventual wound repair
TRIM63	Mediating the glucocorticoid-induced promotion of osteoblastic differentiation

These genes were selected out of 21 HIF-1α target genes in diabetic mice.
